# Comparison of Selected Immune Parameters in a Single Infection and Co-Infection with Infectious Pancreatic Necrosis Virus with Other Viruses in Rainbow Trout

**DOI:** 10.3390/pathogens11060658

**Published:** 2022-06-08

**Authors:** Joanna Maj-Paluch, Magdalena Wasiak, Łukasz Bocian, Michał Reichert

**Affiliations:** 1 Department of Fish Diseases, National Veterinary Research Institute, 57 Partyzantow Avenue, 24-100 Pulawy, Poland; michal.reichert@piwet.pulawy.pl; 2 Department of Pathology, National Veterinary Research Institute, 57 Partyzantow Avenue, 24-100 Pulawy, Poland; magdalena.wasiak@piwet.pulawy.pl; 3 Department of Epidemiology and Risk Assessment, National Veterinary Research Institute, 57 Partyzantow Avenue, 24-100 Pulawy, Poland; lukasz.bocian@piwet.pulawy.pl

**Keywords:** co-infection, apoptosis, propidium iodide

## Abstract

Infectious pancreatic necrosis virus (IPNV) often occurs in an aquatic environment in co-infection with other viruses. In this study, we wanted to investigate the effect of this virus on the course of co-infection with other viruses in rainbow trout. For co-infection we used viral hemorrhagic septicemia virus (VHSV), infectious hematopoietic necrosis virus (IHNV) and salmonid alphavirus (SAV) field strains and infected rainbow trout divided into eight groups; I; IPNV, II; IHNV, III; VHSV, I; SAV, V; IPNV+IHNV, VI; IPNV+VHSV, VII; IPNV+SAV, and the control group. We assessed apoptosis in white blood cells and used a real time RT-PCR to analyze RNA obtained from the internal organs of the fish. During single infection and co-infection the level of expression of immune genes such as interferon and toll-like receptor 3 (TLR-3) was assessed. The highest mortality during the experiment was observed in group III infected by VHSV. The average percentage of apoptotic cells was higher in groups without co-infection, especially in groups II and III. Interferon expression was higher in singly infected groups, the highest being in the heart in group III, while expression of the TLR-3 gene was generally raised in all tested organs in all groups. We found that co-infection with IPNV had a positive impact on the course of infection with the viruses listed because it lowered mortality, reduced apoptosis in co-infected cells, and positively affected fish health.

## 1. Introduction

Infectious pancreatic necrosis virus (IPNV) belongs to the *Birnaviridae* family, and is of the *Aquabirnavirus* genus. The IPNV genome consists of two double-stranded RNA (dsRNA) segments (A and B) which encode structural proteins [1,2]. Infectious pancreatic necrosis virus infections often occur in aquaculture together with those of viruses that cause serious diseases such as viral hemorrhagic septicemia virus (VHSV) [3], infectious hematopoietic necrosis virus (IHNV) [4,5,6], or salmonid alphavirus (SAV) [7]. They can also occur simultaneously with bacterial kidney disease [8]. Co-infection means the simultaneous infection of a host by two or more pathogens at the same time. It is commonplace in aquaculture. Birnavirus–rhabdovirus co-infection of rainbow trout, as described in 1975 by Schlotfeld & Frost, is a good example [9]. IPNV often occurs in salmonids like Atlantic salmon (*Salmo salar*), rainbow trout (*Oncorhynchus mykiss*) or brown trout (*Salmo trutta*), but also can be isolated from any other fish species. The virus is present in both freshwater and marine fish [6,10]. It inflicts 10% minimum mortality but depending on the virulence of the virus strain and amount of the virus, the age of the host, especially in young individuals, and the suitability of the environment, the mortality caused by IPNV may reach 90% [1,11,12,13].

Infections caused by viruses, especially dsRNA viruses like IPNV, affect apoptosis and interferon (IFN) production [14]. During viral infection, the innate immune system recognizes viral pathogens and induces responses protecting the host from infection. This recognitive function is provided by toll-like receptors (TLR), which are transmembrane proteins that recognize pathogen structures and induce the secretion of innate immune effector molecules [15,16]. Toll-like receptor 3 recognizes double-stranded RNA by its ectodomain and triggers downstream signals leading to IFN production [17]. Among the produced interferons, IFN type I is considered to be one of the earliest non-specific anti-viral immune factors to be induced in fish [18,19].

Another important mechanism during viral infection is apoptosis. It is a regulated process of cell death that occurs as a normal part of development [14]. It is also a highly controlled defensive mechanism contributing to the elimination of viral infections by the removal of abnormal or redundant cells. In normal live cells, phosphatidylserine (PS) is located on the cytoplasmic surface of the cell membrane. However, in apoptotic cells, PS is translocated from the inner to the outer surface of the plasma membrane, thus exposing it to the external cellular environment [20]. During leukocyte apoptosis, PS on the outer surface of the cell marks the cell for recognition and phagocytosis by macrophages [21,22].

In this study, rainbow trout were infected with IPNV alone and also with this virus and others simultaneously to induce co-infection and subsequent apoptosis in blood cells. The levels of TLR-3 and IFN type I were checked at specified intervals to characterize differences between single IPNV infection and co-infection with other viruses.

## 2. Results

### 2.1. Disease Symptoms and Lesions

There were no changes in the behavior of rainbow trout from Group I (IPNV) during the experiment. In this group, pale livers and pale and enlarged spleens were observed between 11 and 15 dpi (Table 1).

**Figure 1:**
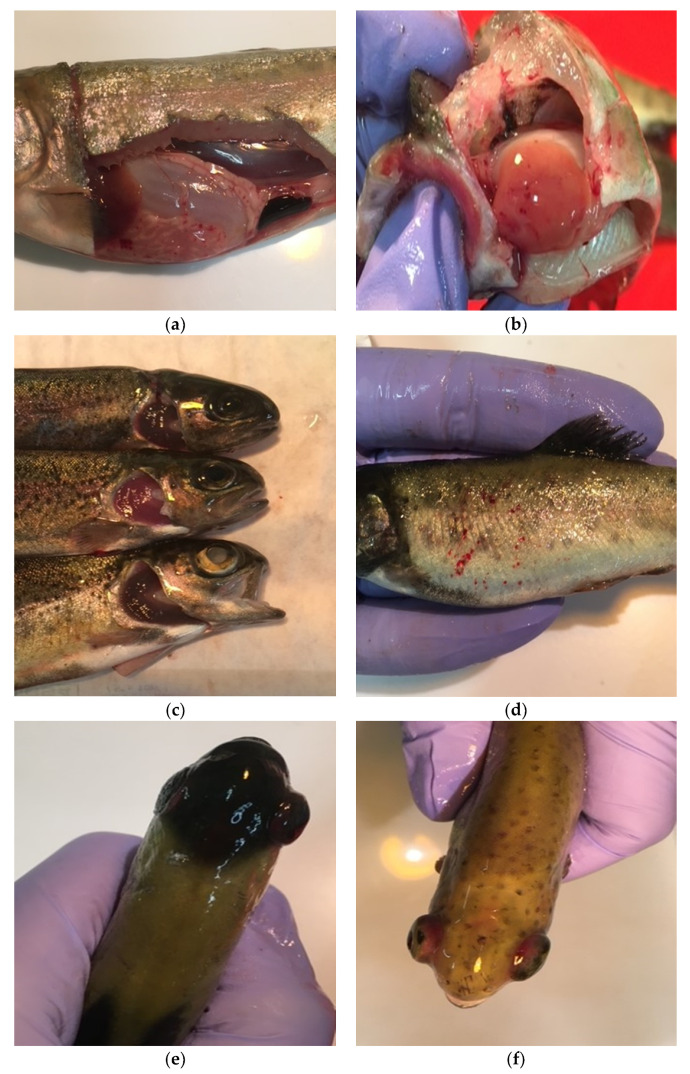
Examples of symptoms observed in fish in Groups II, III, VI during the experiment: (**a**) pyloric caeca (**b**) petechiae on the liver, (**c**) gill necrosis, (**d**) petechiae on the skin, (**e**,**f**) exophthalmia.

**Figure 2:**
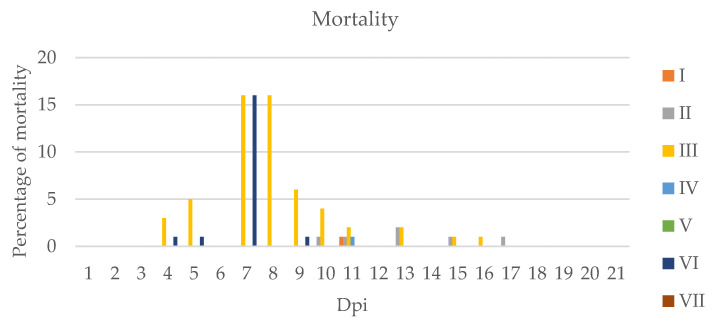
Percentage of mortality observed in groups infected by viruses during the experiment I: group infected with infectious pancreatic necrosis virus (IPNV), II: group infected with infectious hematopoietic necrosis virus (IHNV), III: group infected with viral hemorrhagic septicemia virus (VHSV), IV: group infected with salmonid alphavirus (SAV), V: group infected with IPNV+IHNV, VI: group infected with IPNV+VHSV, VII: group infected with IPNV+SAV, Dpi—day post infection.

In Group II (IHNV), disease symptoms were observed even on the first dpi. During the experiment, symptoms and lesions such as pale livers, liver necrosis, petechiae on the liver and skin, necrosis of the gills and liver and enlarged swim bladders were observed (Figure 1), while in Group V with co-infection (IPNV+IHNV) we observed only petechiae on the liver in some cases. Mortality was observed in Group II, while in Group V all of the fish remained alive (Figure 2).

Although many symptoms of the disease were observed in Group III (VHSV) (Figure 2, Table 1) as early as 2 dpi, the seventh day was the most important for virus replication. The highest mortality in the examined groups was in Group III, with a single VHSV infection. The mortality rate decreased after the ninth dpi. Group VI (IPNV+VHSV) was the only co-infected group in which mortality was observed.

During the experiment, pale livers, petechiae on the skin and enlarged swim bladders and spleens were observed in Group IV (IPNV+VHSV) at 13 dpi. Mortality was approximately 1%. In Group VII (IPNV+SAV), liver necrosis and corkscrew swimming were observed at 18 and 21 dpi and there was no mortality.

Furthermore, during the experiment, differences in blood density were also observed. Blood from fish with visible indicators of the disease was thin, many erythrocytes in it were damaged and it was easy to obtain a lymphocyte layer during the preparation of samples for flow cytometry analysis. Blood from fish without or with only a few lesions, as in Group I or IV, was thick and clotted quickly.

### 2.2. Apoptosis

**Figure 3:**
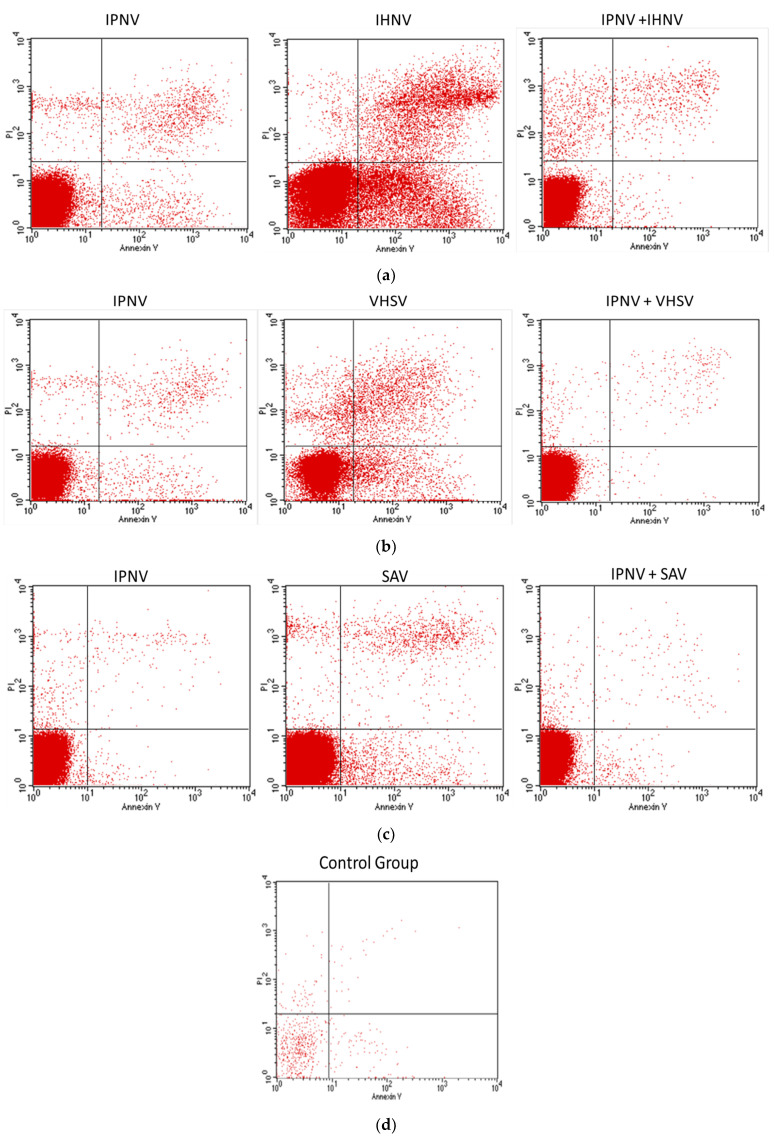
Example comparison of flow cytometry plots of apoptotic cells between a single infection and groups with co-infections showing the control group for reference. The positive effect of IPNV in groups with co-infection is evident. (Y-axis—PI, x-axis—Annexin) (**a**) IPNV, IHNV, and IPNV+IHNV: 18 dpi, (**b**) IPNV, VHSV, and IPNV+VHSV: 21 dpi, (**c**) IPNV, SAV, and IPNV+SAV: 9 dpi, (**d**) Control Group: 3 dpi. Dot plot charts come from a single fish in the group on the particular day post-infection. The average is presented in Figure 4a.

Apoptosis (A) and propidium iodide (PI) labeling data were collected on appropriate days (1, 3, 7, 9, 11, 15, 18, and 21) and the arithmetic average was used for further analysis (Figure 3).

The apoptosis in Group I infected with the IPN virus changed on the 18th day post-infection and began increasing, reaching the highest level of all groups on day 21. 

**Figure 4:**
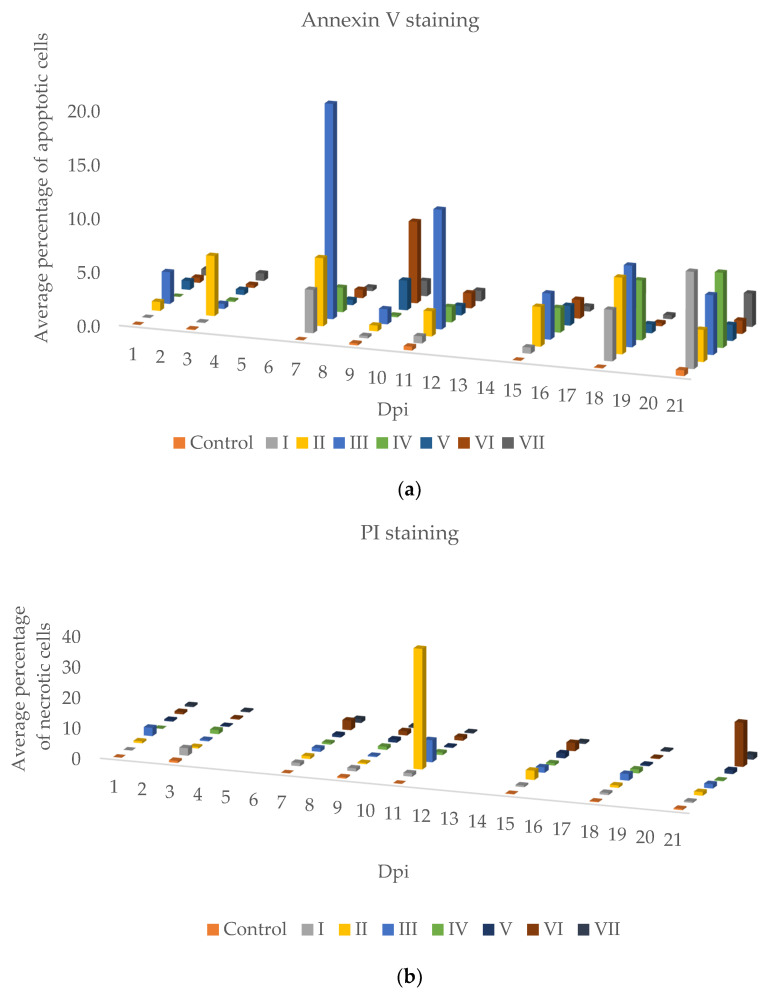
The graph shows: (**a**) the average percentage of apoptotic cells in different groups and on selected days and (**b**) the average percentage of necrotic cells with propidium iodide (PI) in different groups and on selected days. Data were obtained by flow cytometry. Fluorescence excitation for FITC annexin V was 494/518 nm. Fluorescence excitation for PI was 535/617 nm. Control: group not-infected by the virus, I: group infected with IPNV, II: group infected with IHNV, III: group infected with VHSV, IV: group infected with SAV, V: group infected with IPNV+IHNV, VI: group infected with IPNV+VHSV, VII: group infected with IPNV+SAV.

In Group II infected with IHNV, the potentiation of apoptosis was higher than in other groups on the third dpi. After the seventh day, the intensity of apoptosis was less than in other groups, but from the 15th dpi we observed a recovery in apoptosis which remained at a similar level until the end of the experiment. In contrast to Group II, in Group V infected with IPNV+IHNV the level of apoptosis was low during the experiment. The visibility of PI, which is a marker for the number of necrotic cells, was the highest in Group II on the 11th day. In the other groups, PI staining was of low intensity; we only observed an increase in the concentration of this marker in Group VI on the last day of the experiment (Figure 4).

Increases in apoptotic cell percentages were observed in all groups on the seventh day, especially in Group III, which was infected with VHSV (Figure 4). They are related to high mortality on that day in this group. In Group III, an increase in the apoptotic cell percentage was visible even on the first day post-infection and was the highest of all groups during the experiment. In contrast to Group III, in Group VI infected with IPNV+VHSV the increase in the apoptotic cell percentage was observed on the ninth day. Symptoms of the disease were not as numerous as in Group III.

In Group IV infected with SAV, apoptosis remained at a low level, but on the 18th dpi a rise in apoptosis was observed. On day 21 post-infection, the increase in apoptosis was the highest in the whole experiment. Similarly, the increase in apoptosis in Group VII, which was infected with IPNV+SAV, was the greatest on day 21 after infection.

The percentage of apoptotic cells was higher in Groups I–VII than in Group K, but lower than in groups infected with one virus. This observation suggests a protective action of IPNV against other viruses (Figure 4, Supplementary Materials Tables S1 and S2).

### 2.3. IFN, TLR-3

**Figure 5:**
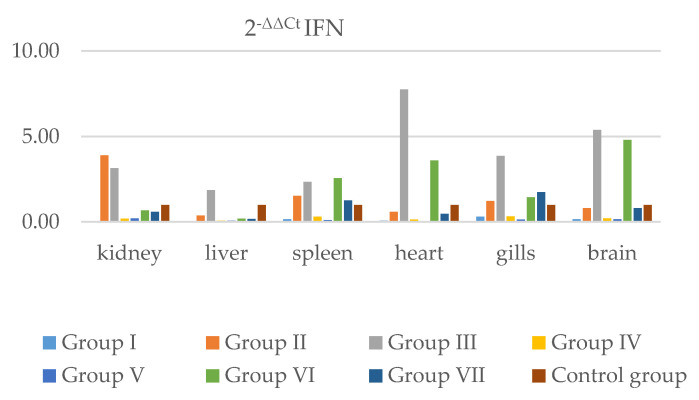
The graph shows relative quantification for the IFN gene in Groups I, II, III, IV, V, VI, and VII and the control group in internal organs, i.e., the kidney, liver, spleen, heart, gills, and brain. Data was collected from all days post-infection.

**Figure 6:**
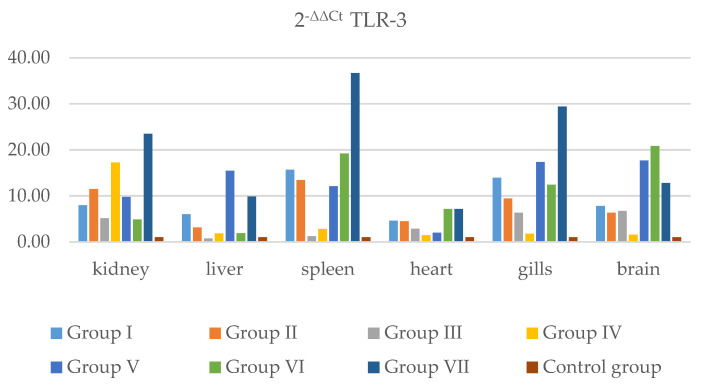
The graph shows relative quantification for TLR-3 in Groups I, II, III, IV, V, VI, and VII and the control group, in internal organs, i.e., the kidney, liver, spleen, heart, gills, and brain. Data was collected from all days post-infection.

The presence of IFN and TLR-3 in individual internal organs was analyzed. The results obtained from real-time RT-PCR were normalized to a housekeeping gene according to the method described by Livak and Schmittgen [23] and Silver et al. [24] Relative differences between the gene of interest (IFN and TLR-3) and the housekeeping gene (ELF-1) were calculated. The 2^−ΔΔCt^ method was used to calculate relative changes in IFN and TLR-3 gene expression (Figure 5, Table S3, Figure 6, Table S4) in the kidney, liver, spleen, heart, gills, and brain. According to the results obtained, the IFN gene in Group I was less strongly expressed than in the control group in all examined organs. The highest expression of IFN in organs was in Groups II and III, where the mortality and apoptosis were higher and clinical symptoms more pronounced than in other groups. In the groups with co-infection with IPNV (V, VI, and VII), a weakening of expression of the IFN gene was observed.

Relative quantification was also calculated to evaluate TLR-3 gene expression in different organs and in each examined group. As we expected, expression of the TLR-3 gene in all organs was higher than in the control group. After infection viral genetic material connects with TLR-3, which induces IFN production. The kidney and spleen are considered to be the most suitable organs for viral replication, and as can be seen, TLR-3 expression was usually higher in these organs than in other organs in all groups.

**Figure 7:**
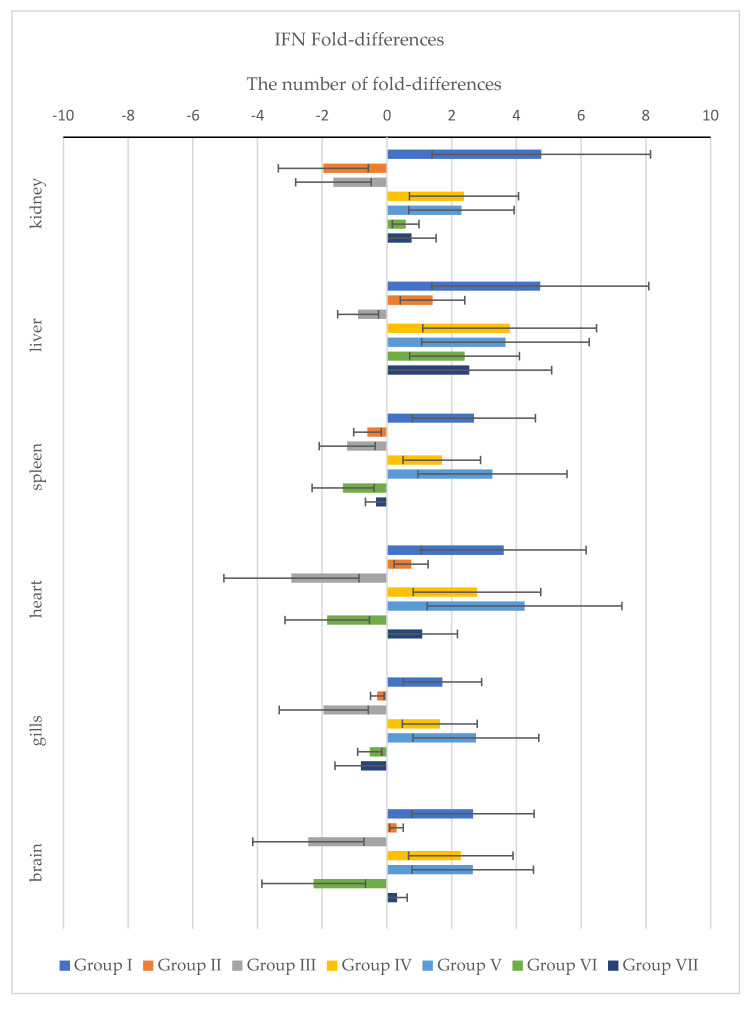
The graph shows fold-differences of the IFN gene in all tested internal organs between the tested groups. All data were normalized to the housekeeping gene ELF1-α and data are presented as fold-change relative. Fold-differences were calculated using the ΔΔCt method and expressed as a range (whiskers: ΔΔCt ± SD) incorporating the standard deviation of ΔΔCt value into the calculation.

**Figure 8:**
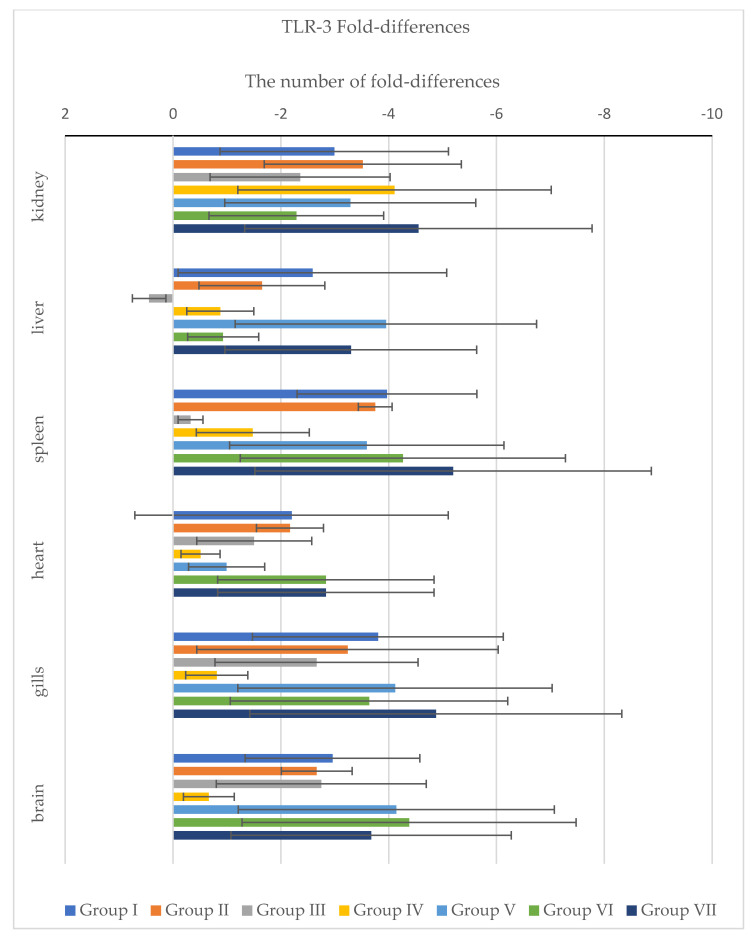
The graph shows fold differences of the TLR-3 gene in all tested internal organs between the tested groups. All data were normalized to the housekeeping gene ELF1-α and data are presented as fold-change relative. Fold-differences were calculated using the ΔΔCt method and expressed as a range (whiskers: ΔΔCt ± SD) incorporating the standard deviation of ΔΔCt value into the calculation.

In addition, standard deviation ΔΔCt and fold-differences for IFN (Figure 7) and TLR-3 (Figure 8) were calculated. According to the obtained results, we can see different expressions between IFN and TLR-3 genes. The main difference is in Group III, where the IFN gene was down-regulated in the kidney, liver, spleen, heart, gills and brain, as well as in Group VI, where this gene was down-regulated in the spleen, heart, gills and brain. A different situation pertained in other groups, where up-regulation was observed.

Down-regulation of the TLR-3 gene was observed in all tested groups and organs, except the liver in Group III, where up-regulation of this gene was noted.

### 2.4. Analysis of Results Obtained by Real-Time RT-PCR

**Figure 9:**
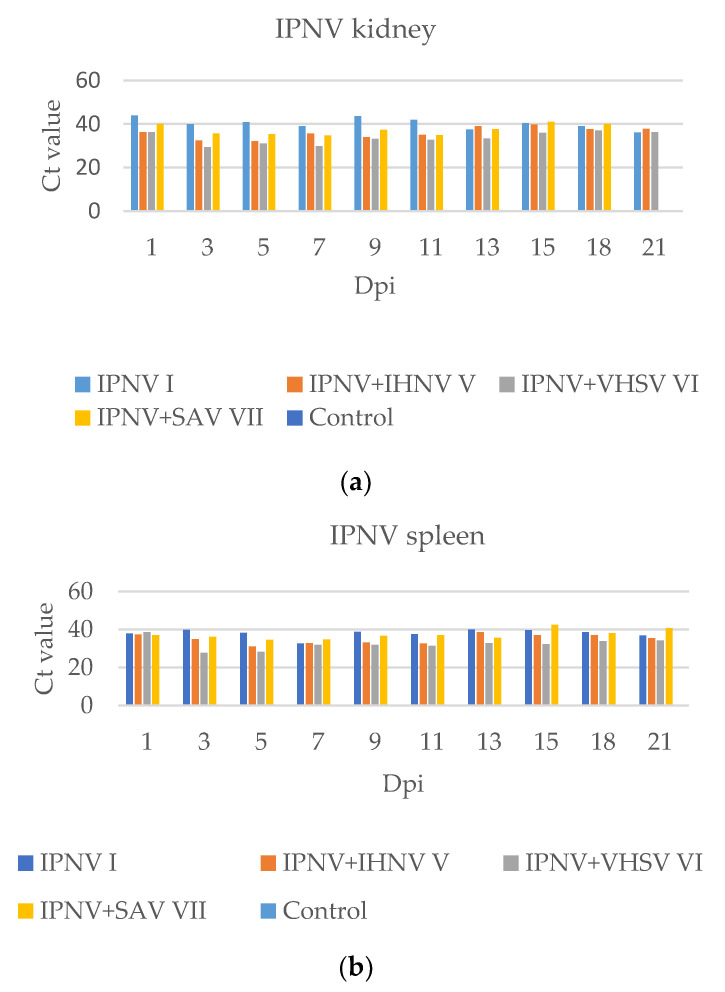
The graphs show the presence of IPNV in the group with single infection (I) and groups with co-infection (V, VI, and VII) in the kidney (**a**), and spleen (**b**), based on the Ct value in a real-time RT-PCR. Where no bar appears for a particular group, no sigmoid curve was generated by the real-time RT-PCR.

Although a high titer of IPNV (1.3 × 10^8^ TCID_50_) was used in the experiment, the presence of the virus was only identified in the kidney and spleen on the first day post-infection (Figure 9). The Ct value of the samples collected from all examined organs in the real-time RT-PCR was rather high during all experiments, which means that IPNV did not replicate quickly. In the groups where IPNV was in co-infection with other viruses, the Ct values were usually lower than that in Group I. However, after 15 dpi a decrease in the titer of IPNV in Group VII compared to those in other groups was observed.

**Figure 10:**
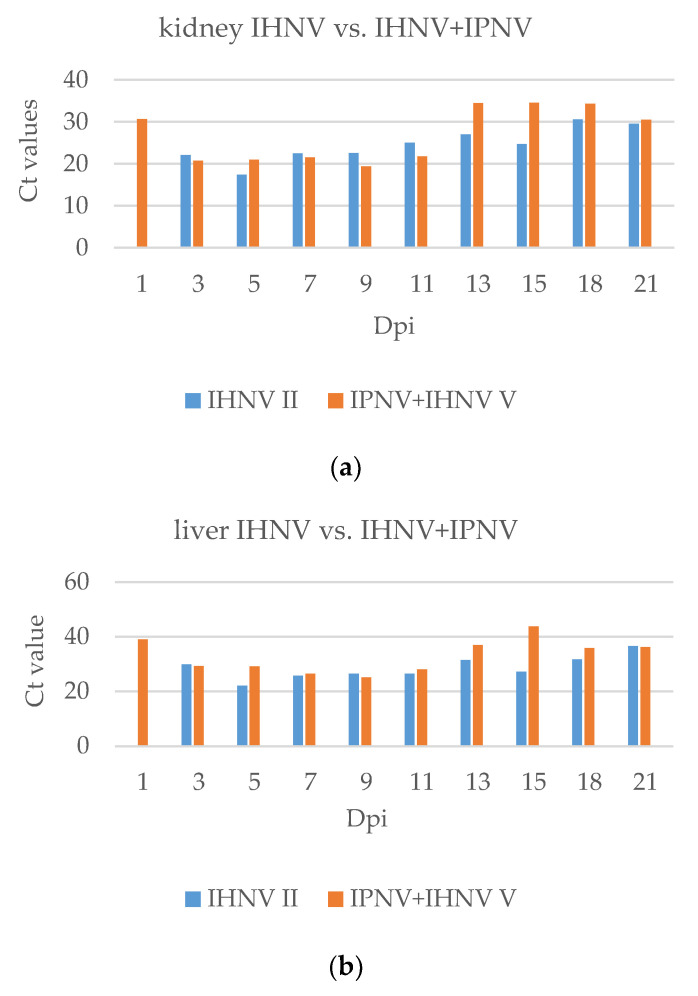
Comparison of the presence of IHNV in the (**a**) kidney and (**b**). liver in single infection and co-infection based on the Ct value in a real-time RT-PCR. Where no bar appears for a particular group, no sigmoid curve was generated by the real-time RT-PCR.

The groups infected with IHNV or IPNV+IHNV (Figure 10) presented divergent results. In general, the Ct values in Group II were lower for all examined internal organs than that in Group V with co-infection. The presence of IHNV in all examined organs of singly infected fish was identified earlier than in organs with co-infection and was noted on the first day. 

**Figure 11:**
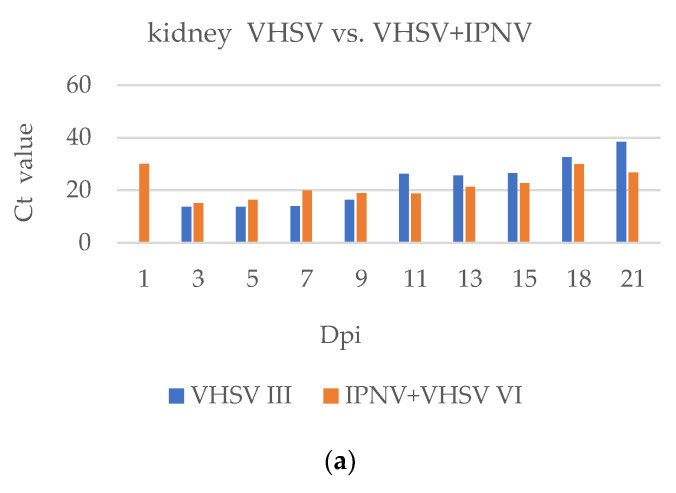

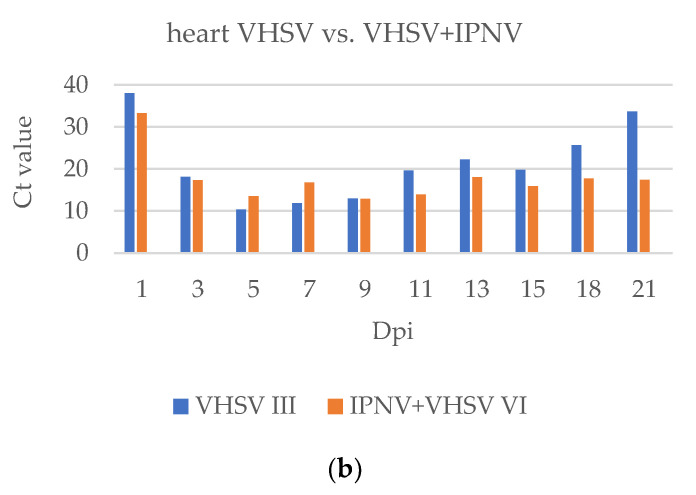
Comparison of the presence of VHSV in the (**a**) kidney and (**b**) heart, in single infection and co-infection based on the Ct value in real-time RT-PCR. Where no bar appears for a particular group, no sigmoid curve was generated by the real-time RT-PCR.

The presence of the VHSV was identified in the co-infected group on the first-day post-infection in all examined organs. In the group with a single infection the presence of VHSV on the first dpi was identified only in the heart (Figure 11). In the early stages of infection (3, 5, 7, and 9 dpi), replication of VHSV was stronger in Group III than in Group VI in all examined organs. After the ninth dpi we observed the opposite and the replication of VHSV was weaker than in Group VI.

**Figure 12:**
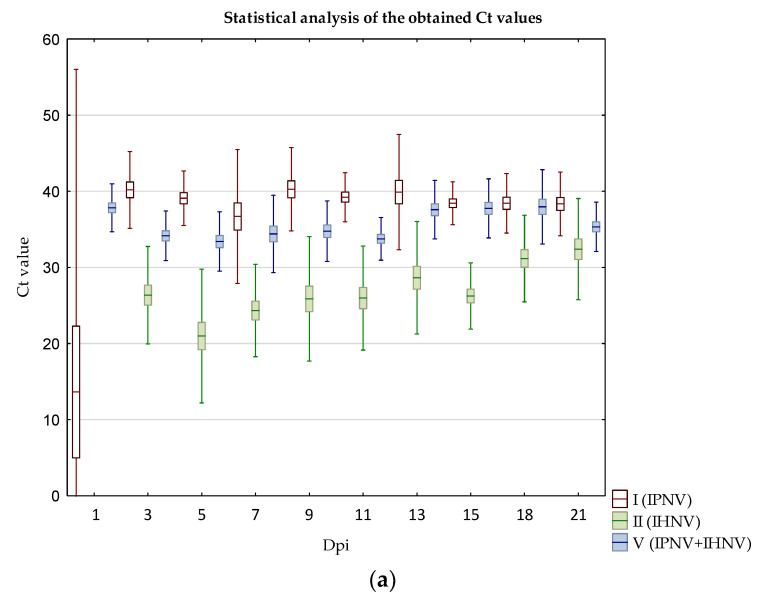

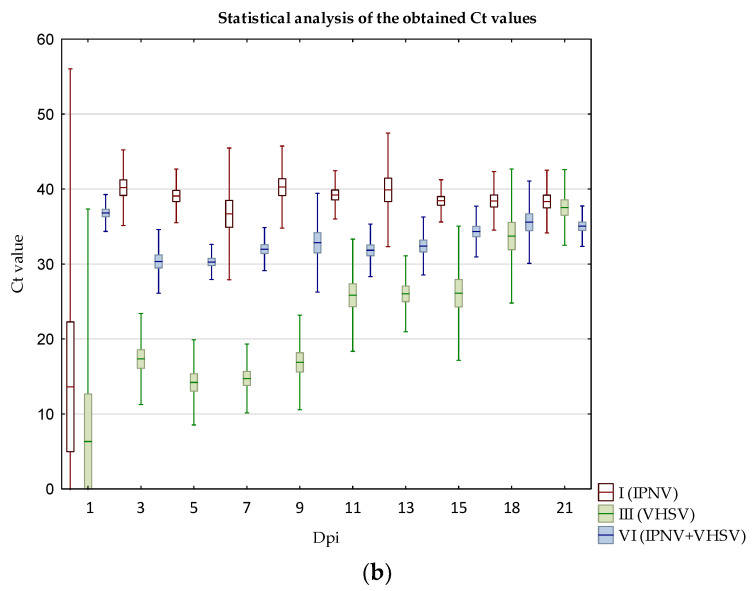
The box plot with statistical comparison results obtained in Real-Time Rt- PCR between groups: (**a**) I (IPNV), II (IHNV), V (IPNV+IHNV), (**b**) I (IPNV), III (VHSV), VI (IPNV+VHSV). The mid-point of the dataset: the average, inter quartile range: the average ± SE, whiskers: the average ± SD. The asterisks present statistically significant differences between groups. (**a**) II (IHNV) and V (IPNV+IHNV), (**b**) III (VHSV) and VI (IPNV+VHSV).

Differences obtained between groups with and without IPNV were tested. Statistically significant results were obtained between groups II (IHNV) and V (IPNV+IHNV) at 1, 3, 5, 7, 9, 11, 13, 15, and 18 dpi and between groups III (VHSV) and VI (IPNV+VHSV) at 1, 3, 5, 7, 9, 11, 13, and 15 dpi (Figure 12). These results confirm that co-infection with IPNV had a positive impact on the course of infection with the IHNV, VHSV.

## 3. Discussion

There is an opinion among trout farmers that the IPNV protects fish against infection with other viruses. This opinion was the reason for undertaking research on IPNV co-infection with other viruses and investigating several factors inherent in the dsRNA of IPNV.

Our results show differences in the average percentage of apoptotic cells between groups infected with one virus and groups with co-infection. Apoptosis in the groups infected with IHNV (II) and VHSV (III) was more intensive than apoptosis in the groups infected with IPNV+IHNV (V) or IPNV+VHSV (VI). Co-infected fish also had less visible lesions. Based on this data, it can be concluded that the IPNV has a positive impact on fish in protecting them from a more severe course of the diseases caused by other viruses. Additionally, in groups with co-infection, mortality was lower. The relative quantification of TLR-3 in different organs is higher in groups with co-infection than in groups with a single infection. This means that the dsRNA of IPNV stimulates the mechanism of induction of TLR-3. It seems the IPN virus also preserved life where other diseases in single infections proved fatal. It could be assumed that the immune system protects cells from apoptosis more effectively by reacting more quickly to the presence of the virus in co-infections than it does in cases of infection caused by one virus. Apoptosis occurs with necrosis, but although the lesions of the disease were observed in liver necrosis or pale livers, breakdown of red blood cells, petechiae, and fluid in the body cavity, this did not correlate with a rise PI staining in cells. The viral infection starts with local invasion, for example of an epithelial surface, and results in the infection of a target organ such as the skin, nervous system, pulmonary tract or parts of the immune system. According to Hwang et al. [25], apoptosis is an active process of cell death that serves diverse functions in multicellular organisms and can provide protection against viral infection by inducing premature death of virus-infected cells. Thus, inside the cells, viruses need to inhibit apoptosis of the host cells in order to replicate efficiently. Failure to inhibit the apoptosis of cells that a virus infects can restrict its growth. A number of viruses depend on the inhibition of apoptosis for normal replication and, consequently, they encode potent cell-death suppressors. These suggestions cohere with our results. IPNV inhibits apoptosis in co-infected cells to keep them alive longer. The lack of apoptosis inhibition in the groups infected only by IHNV or VHSV results in virus growth and consequently cell death.

The immune system plays an important role in the early control of viral infections. According to Barber [14] one of the first lines of defense that confronts an invading virus before it can infect the cells are neutralizing antibodies, which bind to the envelope or capsid proteins of the pathogen and prevent viral attachment and entry into the host [14]. In contrast, Skjesol et al. [26] determined that with type I IFN supplemented to cells after infection with IPNV, those cells are unable to establish a complete antiviral state as efficiently as they would if they had been pre-treated with IFN. This suggests that IPNV has a means to counter cellular defense at stages in the IFN signaling pathways, in addition to being a poor inducer of IFN production. Robertsen [27] suggests, however, that highly virulent virus strains are often more potent inducers of IFNs than low-virulent strains because a virulent virus strain spreads more rapidly throughout the body and encounters many more cells than a non-virulent strain, which is stopped locally at the site of infection. Additionally, different viruses show differences in the induction of IFNs with respect to organs because they have different tissue tropisms [27]. These findings are in accord with our results obtained in different organs and different groups, especially groups with co-infection with IPNV. 

Previous researchers have also found similar results in work concerning the presence of viruses after infection. Alonso et al. [3] described a study on fish singly infected with IPNV or IHNV and co-infected with IPNV and IHNV. They noted lower cumulative mortality in co-infected fish than in the subjects singly infected with IHNV or IPNV. Fish infected with IHNV suffered 92% mortality, and those infected with IPNV 74%, but among fish subjected to co-infection, mortality of only approximately 38% was observed. It may be that the low mortality in fish infected with two viruses is due to genomic variations in genes, which may contribute to the changes observed at the phenotypic level. Authors also observed less IHNV virulence after 45 days in fish infected with two viruses, a possible explanation being the interference by the IPNV with the replication of the IHNV.

Aligned results were obtained by Byrne et al. [7]. They also infected fish with IPNV, IHNV and IPNV+IHNV. High mortality was only observed in the group infected with IHNV. Only 2% of fish died in the co-infected group, giving more foundation to the existence of a protection phenomenon against IHNV infection deriving from the primary IPNV infection. The example findings cited corroborate our results. Fish with IPNV+IHNV or IPNV+VHSV co-infections showed lower mortality than fish with single infections.

Rodriguez et al. [5] also provide information about co-infection with aquabirnaviruses and rhabdoviruses. They conducted an in vitro study and demonstrated that IPNV infection in BF-2 and EPC cell cultures did not influence the replication of the VHSV virus but affected IHNV replication. They applied several methods to evaluate the differences between single infection and co-infection, of which one was flow cytometry, and they quantified viral antigens in cell cultures infected with one or two viruses. They obtained higher percentages of fluorescent cells singly infected with IHNV or VHSV than of such cells co-infected with these viruses and IPNV.

The VHSV and IHNV are known to be dangerous to fish, whereas the IPNV and SAV are known to be less so, the extent depending on the specific variants or other components. Our results prove that the presence of the IPNV in fish cells does not affect the fish’s health dramatically. Instead, in co-infection, it helps carriers to survive, which is implied by the limited symptoms, lower mortality and reduced apoptosis.

Co-infection was also studied by López-Vázquez et al. and mortality reduction was also evident [28]. They studied the effect of IPNV+VHSV co-infection on the mortality caused by both viruses in Senegalese sole. The experiment used an isolate from genogroup I (West Buxton A1 serotype) and showed a 50% increase in the survival rate of co-infected fish over that of fish infected only with VHSV. Pre-exposure to IPNV was found to be responsible for increased Mx expression in superinfected subjects. The authors also observed that in fish that were first exposed to IPNV and then infected with VHSV, both viruses were detected by PCR and recovered from cell culture. In the case of fish first exposed to VHSV and then to IPNV, VHSV was detected in several fish and was not recovered from cell culture, while IPNV was detected by PCR in 83% of fish and recovered from cell culture from all sample pools. They explained this mechanism as the protection conferred by non-specific immune mechanisms on epidermal and epithelial surfaces.

An investigation by de Kinkelin et al. [29] demonstrated that rainbow trout infected first with IPNV and then with VHSV showed a greater ability to survive than fish first infected with VHSV and then with IPNV. They explained this situation as the activation of the IFN system by the first viral infection. Our study does not confirm this theory, because in groups with co-infections the level of IFN was lower than in groups with single infections, where the virus (IHNV or VHSV) spread quickly and caused many symptoms and lesions and inflicted high mortality. A very important factor in viral infection is how virulent the viruses are. Although the titer of IPNV in our experiment was extremely high, the fish were in good health and the IPNV protected them from other virulent viruses. In this study we used the IPNV virus from genogroup V, but López-Vázquez et al. also achieved good results [28] using the virus from genogroup I, and we still do not know what results would be obtained with IPNV from other genogroups with different virulence.

## 4. Materials and Methods

### 4.1. Fish

All procedures carried out on animals were performed in accordance with the local Committee of Ethics on Animal Experimentation (approval number 16/2019).

Rainbow trout (*Oncorhynchus mykiss*) with infectious disease-free status (IPNV, VHSV, IHNV, and SAV) were purchased from a Polish fish farm and were used in this study. The weight of the fish was in the 12–30 g range. They were kept in 200 L tanks with filtered water with a pH of 7 at approximately 10 °C under a light/dark cycle and were allowed to become acclimatized for six weeks prior to the infection. Feeding was daily with suitable pellets for rainbow trout. The composition of the feed was as follows: crude protein 58%, crude fat 17%, NFE 6.0%, ash 10.1%, fiber 0.9%, P 1.2%, gross energy 21.6 MJ, and digestible energy 20.1 MJ. The feed consisted of fish meal, fish oil, functional ingredients, grain products, krill meal, single cell proteins, vegetable proteins, vitamins and minerals. No mortality or changes in fish behavior were observed during the acclimatization period.

Seven hundred and twenty rainbow trout were divided into eight groups and were kept in separate tanks to ensure the right conditions. The control (K) group consisted of 20 fish left free of infection. The other groups consisted of 100 rainbow trout each. The first group (I) was infected with IPNV, the second (II) with IHNV, III with VHSV, IV with SAV, V with IPNV+IHNV, VI with IPNV+VHSV, and the seventh (VII) with IPNV+SAV. To infect fish, subjects from the given group were caught and transferred to a separate smaller tank with water from the large tank and incubated for 1 h with supernatant containing the cell culture–multiplied virus. After that time, fish were transferred back to the large tank. The virus titers were as follows: 1.3 × 10^8^ TCID_50_ for IPNV (serotype A2 Sp, genogroup V), 4.0 × 10^6^ TCID_50_ for IHNV (genotype M), 2.7 × 10^6^ TCID_50_ for VHSV (genotype Ia), and 8.6 × 10^6^ TCID_50_ for SAV (genotype 2). All viruses came from field cases and had been archived at −80 °C. The scheme under which fish were managed is presented in Figure S1.

### 4.2. Flow Cytometry

Rainbow trout blood was collected by puncturing the caudal vein. Ten fish from each virus-infected group were collected on designated days, i.e., at 1, 3, 7, 9, 11, 15, 18 and 21 days post-infection and after virus-directed blood testing; these fish were sources of tissue samples. Blood was diluted by Dulbecco’s phosphate-buffered saline (DPBS), layered carefully onto Lymphoprep density gradient medium for the isolation of mononuclear cells (Stemcell Technologies, Vancouver, BC, Canada) and centrifuged for 25 min at 850× *g*. The density gradient obtained after this step consisted of erythrocytes, a lymphocyte layer and plasma. The lymphocyte layer was collected and washed twice in DPBS. After every dilution, the lymphocytes were centrifuged for 5 min at 3000× *g*. The obtained cells were prepared for flow cytometry according to the manufacturer’s instructions using a FITC Annexin V/Dead Cell Apoptosis Kit with FITC annexin V and PI for Flow Cytometry (Invitrogen, Waltham, MA, USA). Samples were analyzed by flow cytometry in a FACS Calibur machine (Becton Dickinson, Franklin Lakes, NJ, USA). Fluorescence excitation for FITC annexin V was 494/518 nm, and for PI it was 535/617 nm. Kidney, liver, spleen, heart, gill, and brain tissue from five fish were combined into one sample, and the same tissues from another five fish were combined into a second sample and were stored at −80 °C for further analysis. Internal organs but no blood were additionally collected at 5 and 13 dpi. 

### 4.3. Real-Time RT-PCR

Total RNA was extracted from rainbow trout spleen, liver, kidney, heart, gills, and brain tissue derived from Groups I–VII and Group K using a Total RNA Mini Kit (A&A Biotechnology, Gdańsk, Poland), following the manufacturer’s protocol. The samples came from ten fish from Groups I-VII and from two fish from the control group. The RNA pellet was diluted in RNase-free water and the RNA was stored at −80 °C. The concentration and purity were measured using a P330 nanophotometer and 200 ng of RNA was used in each reaction, which was repeated twice. For further analysis, the average Ct value was used.

Identification of IPNV, IHNV, VHSV, and SAV was carried out by using specific primer pairs and probes: IPN-F: 5’-ACC TGC CAT GAA ACC TGA GA-3’; IPN-R: 5’-GGT AAG GGC GTA GCT TCT GA-3’; IPN probe: 5’-FAM-AGC TCC CAC GCC TGT GCA CT-BHQ-1-3’; IHN-F: 5’-AGA GCC AAG GCA CTG TGC G-3’; IHN-R: 5’-TCT TTG CGG CTT GGT TGA-3’; IHN probe: 5’-FAM-TGA GAC TGA GCG GGA CA-BHQ-1-3’; VHS-F: 5’-AAA CTC GCA GGA TGT GTG CGT CC-3’; VHS-R: 5’-TCT GCG ATC TCA GTC AGG ATG AA-3’; VHS probe: 5’-FAM-TAG AGG GCC TTG GTG ATC TTC TG-BHQ-1-3’; SAV-F: 5’-AGA GAT GAT GCC GTT AGC GT-3’; SAV-R: 5’-GAA CTG TTC CCG TGT TAG CC-3’ and SAV probe: 5’-FAM-CCA GCA GCG TGA GCA CGC CC-BHQ-1-3’. Primers and probe sequences for IHNV and VHSV were in line with recommendations included in Commission Implementing Decision (EU) 2015/1554 [30]; there was also a prior source for the sequences for IPNV and SAV because they were based on sequences deposited in GenBank. These primers and probes were designed using GenScript software (GenScript, Piscataway, NJ, USA). The output conditions for IHNV, VHSV, and SAV identification were reverse transcription at 50 °C for 30 min, initial denaturation at 95 °C for 15 min, final denaturation at 94 °C for 15 s, and annealing at 60 °C for 40 s followed by 45 cycles of 10 min at 94 °C and 1 min at 60 °C. The conditions for IPNV identification were reverse transcription at 50 °C for 2 min, initial denaturation at 95 °C for 10 min, final denaturation at 95 °C for 15 s, and annealing at 60 °C for 1 min. Mixtures containing primers, probe and diluted cDNA were then incubated at 95 °C for 15 min, and put through 50 cycles of 10 min at 95 °C and 1 min at 60 °C. The expression of IFN-1 and TLR-3 genes in different rainbow trout tissues was evaluated using real-time RT-PCR. Primers and probes were constructed using GenScript software based on the sequence with GenBank accession number NM_001124578 for TLR-3 and on the sequence with accession number AJ580911.2 for IFN-1. Primer and probe sequences were as follows: TLR-F: 5’-CGA GAG CAT CCT GTG GTT TG-3’, TLR-R: 5’-TCC ATG ATC GAG CGG TTG TA-3’, TLR probe: 5’-FAM-AAA CGC CAG TGT GCC GGG CC-BHQ-1-3’; IFN-F: 5’-ATC CTT GCG GCA CAG ATG T-3’, IFN-R: 5’-CTG ACA GGG TCC CAC ATG AT-3’ and IFN probe: 5’-FAM-CCC AGA CTC ATT TCA GAA GCT ACG CCC-BHQ-1-3’. Two hundred µM of RNA was transcribed into cDNA using a PrimeScript cDNA Synthesis Kit (TaKaRa Bio, Kusatsu, Japan). The real-time PCR was performed with a QuantiTect Probe PCR Kit (Qiagen, Hilden, Germany). The mixture containing primers, probe and diluted cDNA was incubated at 95 °C for 15 min, and then put through 50 cycles of 10 min at 95 °C and 1 min at 60 °C. As an internal control, a pair of primers amplifying the elongation factor ELF1α gene was used. These had the sequences as follows: ELF-1-F: 5’-CCC CTC CAG GAT GTC TAC AAA-3’, ELF-1-R: 5’-CAC ACG GCC CAC GGG TACT-3’, and ELF-1 probe: 5’-HEX-ATC GGC GGT ATT GGA AC-BHQ-1-3’ [31].

### 4.4. Statistical Analysis

The Ct values obtained in the real-time RT-PCR from samples of kidney, liver, spleen, heart, gills, and brain at 1, 3, 5, 7, 9, 11, 13, 15, 18, and 21 dpi were analyzed with Statistica v. 10.0, (StatSoft Polska, Krakow, Poland). Normal distribution was verified using the Shapiro–Wilk test, and homogenity of variance was verified using Levene’s test. A student’s *t*-test was used when distribution was normal and the Mann–Whitney U-test was used when distribution was not normal. 

## 5. Conclusions

In conclusion, we proved in our study that IPNV has a positive impact on rainbow trout health during co-infection with other viruses such as IHNV or VHSV. As a co-infecting agent, IPNV infection in fish diminished the apoptosis of blood cells, made lesions less pronounced, reduced mortality and limited infection to a milder course.

## Figures and Tables

**Table 1:** Symptoms and lesions observed in different groups at 1, 3, 5, 7, 9, 11, 13, 15, 18, and 21 dpi.

DPI	I IPNV	II IHNV	III VHSV	IV SAV	V IPNV+IHNV	VI IPNV+VHSV	VII IPNV+SAV
1		pale liver	pale liver, single petechiae on the skin	Single petechiae on the skin			
3		petechiae on the liver	petechiae on the skin, pale gills and liver, swollen kidney and gut, fluid in the body cavity		petechiae on the liver	petechiae on the gills, pale gills	
5		enlarged swim bladder, swollen kidney	petechiae			pale liver	
7		petechiae on the skin and liver	petechiae on the skin, fluid in the body cavity, petechiae in the eyeball		petechiae on the liver	body swelling, petechiae at the base of the fins, exophthalmia and petechiae in the eyeball, petechiae in the gut, enlarged swim bladder	
9		petechiae on the liver	petechiae on the liver, fluid in the body cavity, petechiae in the eyeball		emergent liver necrosis		
11	pale liver, enlarged kidney	gill and kidney necrosis	enlarged spleen, pale kidney, enlarged swim bladder, petechiae in the muscles and eyeball			fluid in the body cavity, exophthalmia, petechiae in the muscles and gut, gill and kidney necrosis, thin blood	
13	pale liver, enlarged spleen		exophthalmia, petechiae on the skin	pale liver, enlarged swim bladder		fluid in the body cavity, exophthalmia	
15	pale liver		petechiae on the skin	pale liver, enlarged spleen, petechiae on the skin		fluid in the body cavity, exophthalmia, pale liver, gill necrosis	
18		necrotic, marbled liver	pale gills and liver		marbled liver	necrotic liver and gills	changes in the liver
21						pale liver and gills	corkscrew swimming

## Data Availability

Not applicable.

## Supplementary Materials

The following supporting information can be downloaded at: https://www.mdpi.com/article/10.3390/pathogens11060658/s1, Table S1. The average percentage of apoptotic cells in each group on selected days, Table S2. The average percentage of necrotic cells in each group on selected days, Table S3. Results were obtained by calculated relative quantification for IFN gene in Groups I, II, III, IV, V, VI, VII and control group in internal organs: kidney, liver, spleen, heart, gill, brain, Table S4. Results were obtained by calculated relative quantification for TLR-3 gene in Groups I, II, III, IV, V, VI, VII and control group in internal organs: kidney, liver, spleen, heart, gills, brain, Table S5. The standard deviation for IFN ΔΔCt, Table S6. The standard deviation for TLR-3 ΔΔCt.
