# Supplementary material for: Comparison of Selected Immune Parameters in a Single Infection and Co-Infection with Infectious Pancreatic Necrosis Virus with Other Viruses in Rainbow Trout
Source: Pathogens. 2022 Jun 8;11(6):658. doi: 10.3390/pathogens11060658 (PMC9231359; doi:10.3390/pathogens11060658)
Supplement: Supplementary file 1 [file pathogens-11-00658-s001.zip › pathogens-1727409-supplementary.pdf]

Table S1. The average percentage of apoptotic cells in each group on selected days

Dpi	Control	I	II	III	IV	V	VI	VII
1	0,00	0,00	0,832	2,942	0,00	0,828	0,472	0,572
3	0,07	0,066	5,578	0,472	0,126	0,470	0,280	0,692
7	0,01	4,028	6,324	37,675	2,270	0,494	0,768	0,328
9	0,18	0,22	0,536	1,400	0,154	2,750	7,562	1,414
11	0,36	0,668	2,344	11,120	1,422	0,882	1,396	0,980
15	0,01	0,576	3,696	4,286	2,282	1,826	1,740	0,464
18	0,06	4,790	7,128	7,583	5,554	0,832	0,310	0,372
21	0,54	9,032	2,988	5,563	6,988	1,496	1,235	3,094

Table S2. The average percentage of necrotic cells in each group on selected days

Dpi	Control	I	II	III	IV	V	VI	VII
1	0,00	0,00	0,638	2,716	0,00	0,54	0,774	0,612
3	0,68	2,478	0,502	0,466	1,322	0,274	0,39	0,41
7	0,03	1,086	0,91	1,17	0,578	0,854	3,134	1,26
9	0,46	1,02	0,41	0,385	1,034	1,05	1,392	0,546
11	0,06	1,124	39,416	7,278	0,842	0,428	1,216	0,406
15	0,14	0,556	3,063	1,803	0,74	1,684	2,725	0,614
18	0,23	0,708	0,798	2,21	1,318	0,496	0,375	0,408
21	0,42	0,454	1,196	1,437	0,302	1,294	14,545	1,611

Table S3. Results were obtained by calculated relative quantification for IFN gene in Groups I, II, III, IV, V, VI, and VII and control group in internal organs: kidney, liver, spleen, heart, gill, and brain.

	**kidney**	**liver**	**spleen**	**heart**	**gill**	**brain**
Group I	0,04	0,04	0,16	0,08	0,30	0,16
Group II	3,90	0,38	1,52	0,6	1,23	0,81
Group III	3,14	1,86	2,34	7,75	3,87	5,38
Group IV	0,19	0,07	0,31	0,14	0,32	0,21
Group V	0,20	0,08	0,10	0,05	0,15	0,16
Group VI	0,67	0,19	2,56	3,60	1,44	4,80
Group VII	0,59	0,17	1,26	0,47	1,75	0,80
Control group	1	1	1	1	1	1

Table S4. Results were obtained by calculated relative quantification for TLR-3 gene in Groups I, II, III, IV, V, VI, VII and control group in internal organs: kidney, liver, spleen, heart, gill, brain

	**kidney**	**liver**	**spleen**	**heart**	**gill**	**brain**
Group I	7,96	6,01	15,67	4,60	13,95	7,79
Group II	11,47	3,14	13,45	4,49	9,43	6,34
Group III	5,13	0,74	1,25	2,84	6,33	6,73
Group IV	17,26	1,84	2,79	1,43	1,76	1,59
Group V	9,77	15,47	12,08	1,99	17,38	17,67
Group VI	4,89	1,90	19,21	7,14	12,44	20,82
Group VII	23,47	9,85	36,68	7,15	29,41	12,78
Control group	1	1	1	1	1	1

Table S5. The standard deviation for IFN ΔΔCt

	kidney	liver	spleen	heart	gills	brain
Group I	3,372192	3,350979	1,90141	2,551948	1,213395	1,883732
Group II	1,389465	0,997728	0,426385	0,528916	0,210718	0,210011
Group III	1,16814	0,630739	0,866206	2,088793	1,380272	1,716148
Group IV	1,684328	2,68347	1,19996	1,970707	1,157534	1,613618
Group V	1,629174	2,589425	2,306582	3,009446	1,943129	1,876661
Group VI	0,412243	1,697056	0,95813	1,305319	0,373352	1,600183
Group VII	0,537401	1,800294	0,235467	0,771453	0,568514	0,221324

Table S6. The standard deviation for TLR-3 ΔΔCt

	kidney	liver	spleen	heart	gills	brain
Group I	2,116371	1,829285	2,807214	1,556342	2,68842	2,093743
Group II	2,489016	1,166019	2,650943	1,533008	2,289612	1,883732
Group III	1,668065	0,311834	0,231224	1,064903	1,882318	1,945251
Group IV	2,905502	0,622254	1,045811	0,362039	0,576292	0,47164
Group V	2,325674	2,793779	2,542049	0,702864	2,912573	2,929543
Group VI	1,618567	0,656902	3,015103	2,004648	2,571747	3,097128
Group VII	3,219457	2,333452	3,674834	2,006062	3,449267	2,599325
